# Transcriptomic analysis reveals inhibition of androgen receptor activity by AMPK in prostate cancer cells

**DOI:** 10.18632/oncotarget.1997

**Published:** 2014-05-21

**Authors:** Sarah Jurmeister, Antonio Ramos-Montoya, David E. Neal, Lee G D Fryer

**Affiliations:** ^1^ Uro-Oncology Research Group, Cancer Research UK Cambridge Institute, University of Cambridge, Li Ka Shing Centre, Robinson Way, UK; ^2^ Dept of Urology, University of Cambridge, S4, Dept of Oncology, Addenbrooke's Hospital, UK

**Keywords:** Androgen receptor, AMPK, prostate cancer, cancer metabolism, transcription

## Abstract

Metabolic alterations contribute to prostate cancer development and progression; however, the role of the central metabolic regulator AMP-activated protein kinase (AMPK) remains controversial. The androgen receptor (AR), a key driver of prostate cancer, regulates prostate cancer cell metabolism by driving the expression of a network of metabolic genes and activates AMPK through increasing the expression of one of its upstream kinases. To more clearly define the role of AMPK in prostate cancer, we performed expression profiling following pharmacologic activation of this kinase. We found that genes down-regulated upon AMPK activation were over-expressed in prostate cancer, consistent with a tumour suppressive function of AMPK. Strikingly, we identified the AR as one of the most significantly enriched transcription factors mediating gene expression changes downstream of AMPK signalling in prostate cancer cells. Activation of AMPK inhibited AR transcriptional activity and reduced androgen-dependent expression of known AR target genes. Conversely, knock-down of AMPK increased AR activity. Modulation of AR expression could not explain these effects. Instead, we observed that activation of AMPK reduced nuclear localisation of the AR. We thus propose the presence of a negative feedback loop in prostate cancer cells whereby AR activates AMPK and AMPK feeds back to limit AR-driven transcription.

## INTRODUCTION

Prostate cancer is the third leading cause of cancer death in developed countries and the most common cancer in men [1]. The androgen receptor (AR) plays a key role in both normal prostate biology and prostate cancer progression and, as a result, targeting of AR signalling is a major therapeutic strategy for advanced prostate cancer [2]. While initial response rates to androgen deprivation therapy (ADT) are high, therapeutic options for castration resistant disease are limited. Notably, castration-resistant prostate cancer (CRPC) remains dependent on AR signalling, but becomes insensitive to ADT through a variety of mechanisms [3-8].

During recent years, the observation that cancer cells frequently display an altered metabolism has gained increasing attention, to the point where metabolic deregulation is now considered one of the emerging hallmarks of cancer, and strong evidence suggests that metabolic alterations also play an important role in prostate cancer [9, 10]. Intriguingly, recent studies have unveiled a high degree of crosstalk between AR signalling and metabolic pathways in prostate cancer cells. The AR has been shown to regulate prostate cancer metabolism by driving the expression of an extensive network of metabolic genes, such as fatty acid synthase and alpha-methylacyl-CoA racemase, resulting in stimulation of both aerobic glycolysis and anabolic pathways [11-17]. Another gene whose expression is driven by the AR is Calcium/Calmodulin-Dependent Protein Kinase Kinase 2 (*CAMKK2*), one of the upstream kinases of the metabolic master regulator AMPK. AMPK is a highly conserved heterotrimeric serine/threonine kinase consisting of a catalytic alpha and regulatory beta and gamma subunits [18, 19]. It acts as an energy sensor that is activated in response to numerous stress factors that decrease cellular ATP:AMP ratio, such as hypoxia or glucose deprivation, and facilitates the restoration of cellular energy balance by activating catabolic pathways and inhibiting anabolic pathways. In prostate cancer cells, AR-driven up-regulation of *CAMKK2* has been shown to result in increased AMPK activity upon androgen stimulation [11, 20]. This mechanism has been proposed to drive prostate cancer cell growth through multiple mechanisms, including increased glycolysis and mitochondrial biogenesis [11, 21].

Despite a large number of studies investigating AMPK function, its role in prostate cancer remains controversial. Several lines of evidence suggest that AMPK has tumour suppressor properties in the prostate. Knock-out of its upstream kinase *LKB1* results in prostatic intraepithelial neoplasia (PIN) in mouse models [22]. Moreover, retrospective studies suggest that metformin, a commonly used anti-diabetic drug that is thought to partially act through activation of AMPK, may decrease the risk of developing various types of cancer, including prostate [23-25]. In line with this, pharmacologic activation of AMPK has been reported to decrease growth and viability of several prostate cancer cell lines *in vitro* and to inhibit lipid synthesis induced by the synthetic androgen R1881 [26]. Multiple mechanisms may contribute to these effects of AMPK, including inhibition of the mTOR pathway, up-regulation of p53 and p21 [27] and induction of apoptosis through generation of reactive oxygen species as well as through cross-talk with TNF signalling [28, 29]. In addition to its anti-proliferative effects, AMPK may also enhance the sensitivity of cancer cells to therapeutic treatments. For example, metformin has been shown to sensitise cancer cells to chemotherapy while exerting protective effects on normal epithelial cells [30]. Similarly, AMPK activation can sensitise cancer cells to ionizing radiation [31] and to treatment with the multi-tyrosine kinase inhibitor dasatinib [32]. Finally, the growth inhibitory effects of adiponectin and a number of natural compounds on prostate cancer cells have been at least partially attributed to their ability to activate AMPK [33-35]. However, other studies suggest that AMPK could have tumour-promoting functions in the prostate and that AMPK activation is higher in prostate cancer than in normal tissue [21, 36]. Indeed, it has been proposed that AMPK may actually contribute to resistance to anti-cancer therapy in some settings [37-39].

Intriguingly, metformin has been reported to not only reduce the risk of cancer, but of other age-related diseases, opening up possibilities for novel applications of this well-established drug [40, 41]. Taken together, these findings have sparked considerable interest both in the development of novel drugs targeting AMPK and related pathways in a clinical setting, and in a potential use of existing AMPK activators such as metformin in the treatment of cancer and other diseases.

In light of the contradictory findings on the role of AMPK in cancer, we sought to improve our understanding of this signalling pathway in prostate cancer cells by characterising the transcriptional output of AMPK activation. Genome-wide expression profiling revealed that AMPK signalling resulted in the repression of genes that are commonly overexpressed in prostate cancer. Intriguingly, the AR was identified as a potential downstream mediator of AMPK signalling. We then went on to show that activation of AMPK decreased AR activity and nuclear localisation, suggesting the presence of a negative feedback loop between AMPK and AR in prostate cancer cells. Our study thus provides a novel mechanism of cross-talk between these two major metabolic drivers in prostate cancer cells.

## RESULTS

### Transcriptional impact of AMPK activation in prostate cancer cells

**Figure 1:**
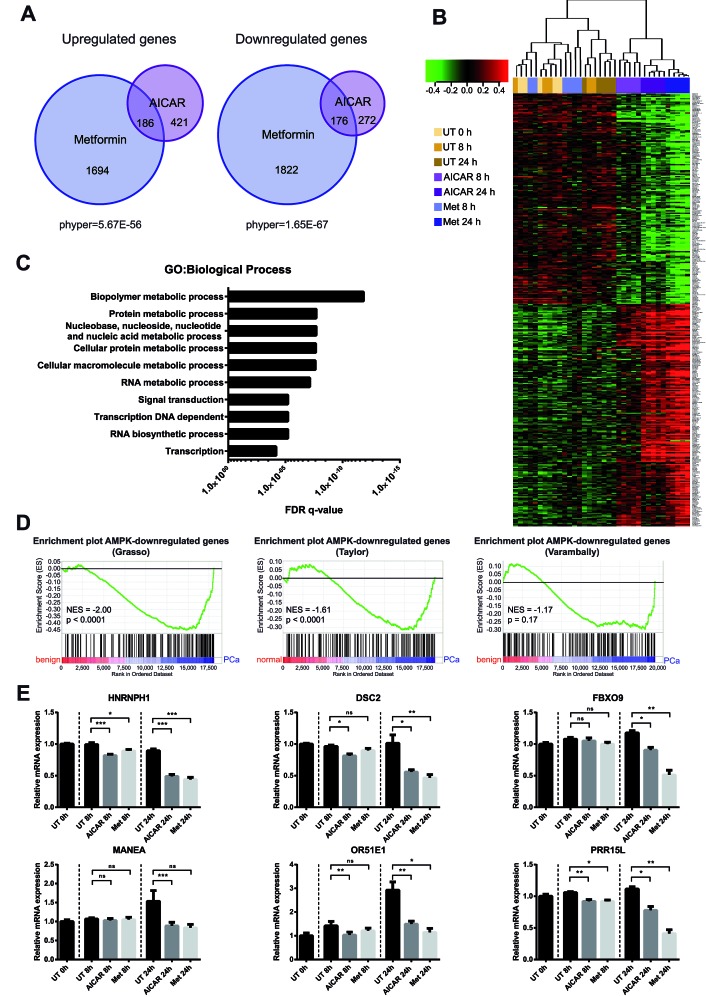
Genome-wide expression profiling reveals the transcriptional impact of AMPK activation in prostate cancer cells A: LNCaP cells were treated with AICAR or metformin for 8 h and 24 h. Gene expression was analysed using Illumina Humanv4 BeadChip arrays. Differentially expressed genes (DEG's) were identified using a globally applied FDR-corrected p-value cut-off of 0.01 and compared between treatments. The overlap between gene lists is depicted as an area-proportional Venn diagram. Hypergeometric test was performed to test significance of overlaps between treatments. B: Heatmap showing expression of 362 putative AMPK-regulated genes. Samples were clustered according to Pearson correlation. C: Biological processes enriched among 362 putative AMPK target genes according to gene ontology terms. D: Enrichment plots of AMPK-repressed genes in published prostate cancer datasets. Genes in the Grasso, Taylor and Varambally datasets were ranked according to their expression in benign/normal prostate tissue compared to primary prostate cancer. Gene set enrichment analysis (GSEA) was used to determine enrichment of AMPK-repressed genes within these phenotypes. NES: normalised enrichment score. E: qRT-PCR validation of selected AMPK-repressed genes that are overexpressed in primary prostate cancer. n=6.

We used genome-wide expression profiling to determine differentially expressed genes in the LNCaP cell line upon AMPK activation. To take account of potential off-target effects of AMPK activating drugs, we used two structurally unrelated, commonly used AMPK activators, 5-aminoimidazole-4-carboxamide ribonucleotide (AICAR) and metformin [42, 43], and compared their transcriptional effects in prostate cancer cells. After 8 h, we identified 489 differentially expressed genes following AICAR treatment, but only 41 after treatment with metformin (FDR 0.01; Table 1). In contrast, 711 and 3864 genes were differentially expressed after 24 h of treatment with AICAR or metformin, respectively. We compared up- and down-regulated genes between treatments irrespective of time point and found a highly significant overlap between the two AMPK activators (Figure 1A). Approximately 65% of AICAR- and 90% of metformin-regulated genes were not shared between treatments. As differences in the gene expression profiles of these two drugs could potentially represent AMPK-independent effects, we focussed our downstream analysis on the 362 genes that were concordantly regulated by both drugs and were thus more likely to be AMPK-regulated (Figure 1B). In line with the established role of AMPK as a metabolic master regulator, biological processes enriched among these 362 genes were predominantly metabolism-related (Figure 1C). Using Connectivity Map 02 (http://www.broadinstitute.org/cmap/↱;[44]) to compare our AMPK signature to a collection of gene-expression signatures associated with 1,309 bioactive small molecules we found that our gene set closely resembled signatures of three compounds known to inhibit the PI3K/Akt/mTOR pathway (thioridazine, wortmannin and LY-294002; Supplementary Table 1). In accordance with this, inhibition of mTOR is one of the best-described functions of AMPK [45-47]. Our set of 362 putative AMPK target genes was thus consistent with the established biological function of AMPK.

**Table 1:** Differentially expressed genes in prostate cancer cells following AMPK activation LNCaP cells were treated with AICAR or metformin for 8 h and 24 h. Gene expression was analysed using Illumina Humanv4 BeadChip arrays. Numbers of differentially expressed genes (DEG's) identified using a globally applied FDR-corrected p-value cut-off of 0.01 are shown

Time point	AICAR	Metformin
8 h	489	41
24 h	711	3864

To investigate whether AMPK-regulated genes might have a role in prostate cancer, we used gene set enrichment analysis (GSEA) [48, 49] to compare our set of AMPK target genes to three published expression datasets comparing benign/normal prostate tissue with primary and metastatic prostate cancer (Grasso, Taylor and Varambally datasets) [50-52]. Strikingly, genes that were down-regulated upon AMPK activation were significantly enriched among genes over-expressed in primary prostate cancer, compared to normal/benign tissue, in both the Grasso and Taylor datasets (NES=−2.00 and NES=−1.61, respectively). A similar trend was observed in the Varambally dataset (NES=−1.17), although it did not reach statistical significance. AMPK-induced genes did not show significant enrichment in any of the three datasets.

We then selected six genes (*HNRNPH1*, *DSC2*, *FBXO9*, *MANEA*, *OR51E1*, *PRR15L*) for validation by qRT-PCR that were over-expressed in prostate cancer across all datasets and showed high fold changes in our microarray (Supplementary Figure 1B). To our knowledge, none of these genes have previously been shown to be AMPK regulated. Of note, one of the selected genes, *OR51E1*, has been proposed as a prostate cancer biomarker [53-55] and formed part of the core enrichment in all three GSEA analyses (Supplementary Figure 1C). Reduced expression of all six genes upon AICAR or metformin treatment was confirmed by qRT-PCR (Figure 1E). Together, these results showed that activation of AMPK resulted in reduced expression of genes that are associated with primary prostate cancer.

### The AR is a candidate transcription factor acting downstream of AMPK in prostate cancer cells

**Figure 2:**
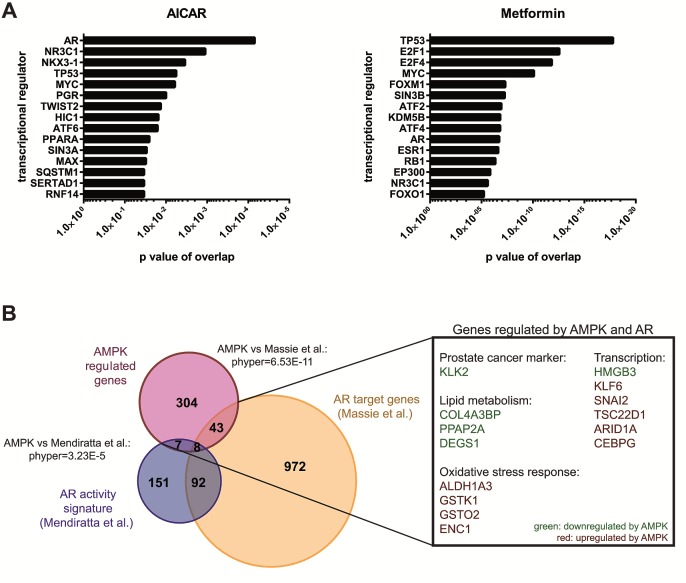
The androgen receptor is a potential transcription factor downstream of AMPK A: Ingenuity Pathway analysis was used to predict significantly enriched transcriptional regulators among differentially expressed genes following 24 h of AICAR or metformin treatment. B: AMPK target genes were compared to two published datasets describing AR target genes [11, 60]. The overlap between gene lists is depicted as an area-proportional Venn diagram. Hypergeometric test was performed to test significance of overlaps between treatments. Examples of genes regulated by AMPK and by AR in at least one dataset are shown.

To identify pathways mediating the transcript-level effect of AMPK activation in prostate cancer cells, we used Ingenuity Pathway Analysis (IPA) to predict potential transcriptional regulators of the AICAR- and metformin-regulated genes in our dataset (Figure 2A). Comparing the fifteen most significantly enriched transcriptional regulators for each treatment, we found four that were shared between both treatments: AR, p53, glucocorticoid receptor (*NR3C1*) and *MYC*. Regulation of p53 [56] and *NR3C1* [57] by AMPK has been previously demonstrated, confirming that our approach was able to identify established AMPK-regulated transcriptional pathways in prostate cancer cells. After AICAR treatment, the AR was the most significantly enriched transcriptional regulator, and *NKX3.1*, a well-established AR regulated gene [58, 59], was ranked third. The enrichment after treatment with metformin was not as striking, as the AR was only ranked tenth in this condition. However, we still found a highly significant overlap between metformin- and androgen receptor regulated genes (Figure 2A).

This finding, together with the central role of AR in prostate cancer development and progression, prompted us to further investigate the potential link between AMPK signalling and AR activity. We thus compared our subset of putative AMPK target genes to previously published data on AR target genes [11] as well as a published AR activity signature [60] and confirmed significant overlap between AMPK- and AR-regulated genes (Figure 2B). Specifically, 16% of AMPK regulated genes also showed evidence of being AR-regulated in at least one of the two datasets. Of note, among the genes repressed by AMPK was *KLK2*, a prostate cancer marker and a well-established AR target gene that belongs to the same gene family (kallikrein-related peptidases) as prostate specific antigen (PSA / *KLK3*). Additionally, a number of genes involved in lipid metabolism (*DEGS1*, *PPAP2A*, *COL4A3BP*) that were induced by AR were also down-regulated by AMPK, consistent with the previously-established roles of both of these proteins in the regulation of lipid metabolism. Taken together, these findings led us to focus on the AR as a potential downstream effector of AMPK signalling.

### Activation of AMPK inhibits the transcriptional activity of AR

**Figure 3:**
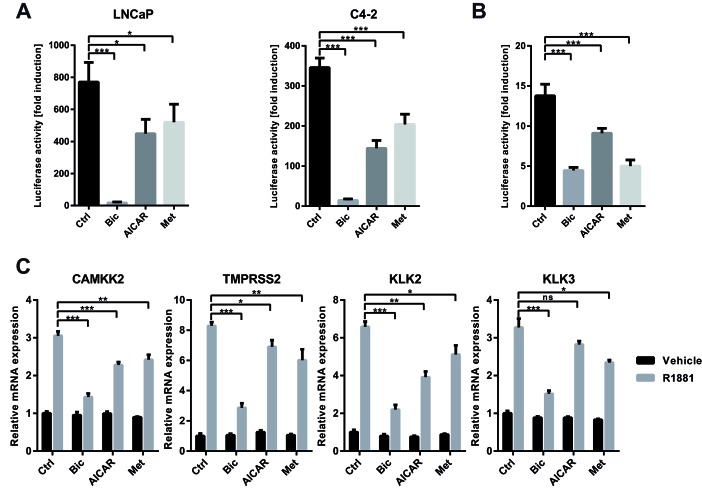
Activation of AMPK inhibits the transcriptional activity of AR A: An androgen-responsive dual luciferase reporter assay was used to assess effects of AMPK activation on AR activity. LNCaP and C4-2 cells were transfected with MMTV-*Luc* and pRLTK and treated with R1881 and bicalutamide, AICAR or metformin for 24 h. Fold induction of luciferase activity was calculated with respect to a control without R1881. n=3. B: LNCaP cells were stably transduced with an androgen-responsive luciferase reporter derived from the *CAMKK2* promoter. Cells were treated with R1881 and bicalutamide, AICAR or metformin for 24 h. Fold induction of luciferase activity was calculated with respect to a control without R1881. n=5. C: To assess effects of AMPK activation on induction of endogenous AR-target genes by R1881, C4-2 cells were grown in androgen-free medium for three days and stimulated with R1881 and bicalutamide, AICAR or metformin for 12 h. mRNA expression was assessed by qRT-PCR; values are depicted relative to vehicle control. n=4 for *CAMKK2* and *KLK2*, n=3 for *KLK3* and *TMPRSS2*.

To determine if AMPK activation regulates androgen receptor-driven transcription, we employed an androgen-responsive luciferase reporter system (MMTV-*Luc*). Treatment of transiently transfected LNCaP cells with the synthetic androgen R1881 resulted in an approximately 800-fold increase in luciferase activity compared to vehicle control, an induction that could be almost completely abrogated by the anti-androgen bicalutamide. Importantly, co-treatment with either AICAR or metformin resulted in a significant decrease in luciferase induction compared to R1881 alone, suggesting that activation of AMPK does indeed modulate AR activity (Figure 3A). We obtained similar results in the castrate-resistant LNCaP derived cell line C4-2, suggesting that the negative regulation of AR by AMPK is maintained in this model of castration-resistant prostate cancer. To further confirm these findings, we created a second androgen-responsive luciferase reporter containing an androgen-responsive element derived from the promoter of *CAMKK2* and introduced it in a lentiviral vector in order to generate stable AR-reporter cell lines. In LNCaP cells stably transduced with the *CAMKK2* reporter, AICAR and metformin significantly decreased R1881-induced luciferase activity, confirming the inhibitory effect of AMPK activation on AR activity (Figure 3B).

We then tested the effect of AMPK activation on the androgen-mediated induction of endogenous AR target genes (Figure 3C). In C4-2 cells, treatment with AICAR or metformin significantly attenuated induction of established AR target genes *CAMKK2*, *KLK2*, and *TMPRSS2* by R1881 (Figure 3C). Induction of *KLK3* (PSA) was also attenuated, but only reached significance with metformin. Comparable results were obtained in LNCaP cells (Supplementary Figure 2). In summary, these findings demonstrate that activation of AMPK negatively regulates AR transcriptional activity.

### Silencing of AMPK catalytic subunits stimulates the transcriptional activity of AR

**Figure 4:**
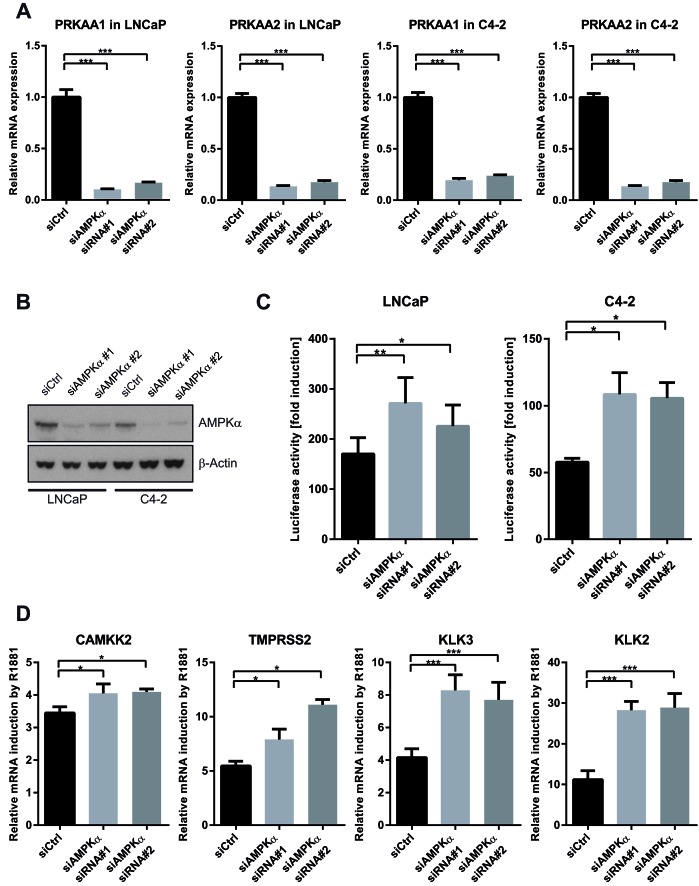
Silencing of AMPK increases the transcriptional activity of AR A: LNCaP and C4-2 cells were transfected with two different combinations of siRNAs directed against the AMPKα isoforms (*PRKAA1* and *PRKAA2*). Successful knock-down was validated using qRT-PCR three days after transfection. n=4. B: LNCaP and C4-2 cells were transfected with two different combinations of siRNAs directed against both AMPKα isoforms. Successful knock-down was validated by Western Blot four days after transfection. C: An androgen-responsive dual luciferase reporter assay was used to assess effects of AMPK knock-down on AR activity. LNCaP or C4-2 cells were co-transfected with MMTV-*Luc*, pRL-TK and siRNAs for 24 h and treated with R1881 for 40 h. Fold induction of luciferase activity was calculated with respect to vehicle control for each siRNA. n=5 for LNCaP cells, n=4 for C4-2 cells. D: To assess effects of AMPK knock-down on induction of endogenous AR-target genes by R1881, LNCaP cells were transfected with siRNAs and grown in androgen-free medium for three days, then stimulated with R1881 for 12 h. mRNA expression was assessed by qRT-PCR; values are depicted relative to vehicle control for each siRNA. n=4, except for *TMPRSS2*: n=3.

To further demonstrate that the effects of AICAR and metformin on AR activity are mediated through AMPK, we made use of two different combinations of siRNAs directed against the catalytic AMPKα subunits to achieve good levels of knock-down for both isoforms, AMPKα1 (*PRKAA1*) and AMPKα2 (*PRKAA2*) (Figures 4A and B). Silencing of AMPKα resulted in an increased activation of AR by R1881 in both LNCaP and C4-2 cells (Figure 4C) and an increased androgen-induced expression of the AR target genes *CAMKK2*, *KLK2*, *KLK3* and *TMPRSS2* (Figure 4D). These data ruled out that results observed from pharmacological activation of AMPK were off-target effects and further supported the finding that AMPK is a negative regulator of AR.

### Activation of AMPK reduces nuclear localisation of AR

**Figure 5:**
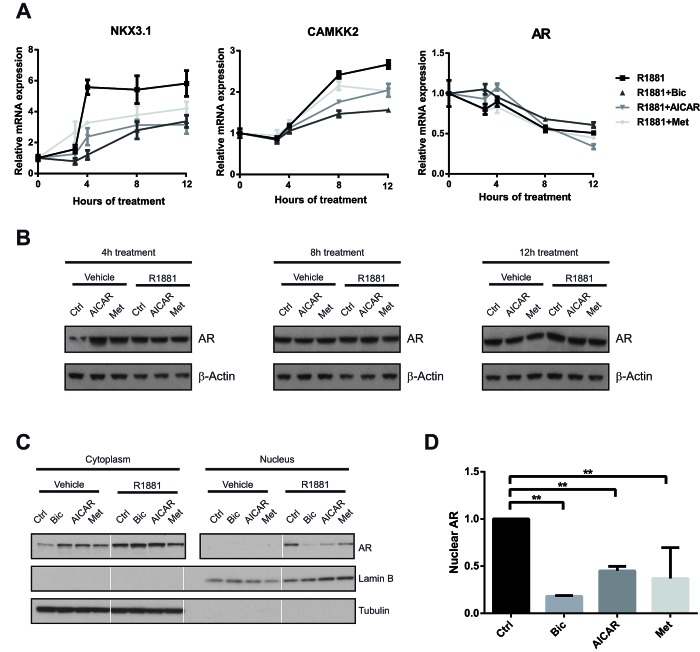
Activation of AMPK reduces nuclear localisation, but not expression of AR A: To assess the dynamics of AR inhibition following AMPK activation, LNCaP cells were grown in androgen-free medium for three days, then stimulated with R1881 and bicalutamide, AICAR or metformin for 3, 4, 8 and 12 h. Expression of *NKX3.1*, *CAMKK2* and AR was quantified by qRT-PCR. n=3. B: To evaluate effects of AICAR and metformin on AR protein level, LNCaP cells were grown in androgen-free medium for three days, then stimulated with R1881 and bicalutamide, AICAR or metformin for 4, 8 and 12 h. AR protein levels were assessed by Western Blot. C: To assess effects of AICAR and metformin on localisation of the AR, C4-2 cells were grown in androgen-free medium and stimulated with drugs as indicated for 12 h. Cytoplasmic and nuclear fractions were separated, and AR protein levels were assessed by Western Blot. Lamin B and tubulin were used as loading controls and to confirm successful fractionation. White lines separate non-contiguous bands run on the same gel. D: Nuclear AR levels after androgen stimulation were quantified using Western Blots from three independent experiments and normalised to the Lamin B signal.

Two previous studies had reported that metformin treatment of prostate cancer cells down-regulates the expression of AR, though neither of them addressed potential effects on the expression of androgen-regulated genes [61, 62]. To test whether this mechanism could explain our finding that AMPK activation inhibits AR activity, we investigated the dynamics of AR inhibition by AICAR and metformin in more detail. Notably, in previous studies, reduction of AR expression was observed after relatively long metformin treatments (96 h) when used at comparable concentrations to our own study, whereas we observed decreased induction of AR-regulated genes already at 12 h (Figure 3C). We performed a time course of the expression of *CAMKK2* and another established AR target gene, *NKX3.1*, which was chosen due to its rapid response to androgen, after co-treatment with R1881 and AICAR or metformin (Figure 5A). In both cases, the inhibitory effects of AICAR and metformin on the expression of these genes became apparent as soon as appreciable R1881-induced induction was observed (four and eight hours for *NKX3.1* and *CAMKK2*, respectively). *AR* mRNA was slightly decreased by all treatments, consistent with previous reports that androgens reduce *AR* mRNA levels [63, 64], but no further decrease was observed following AICAR or metformin treatment. More importantly, there was no appreciable decrease in AR protein level with AICAR or metformin treatment during this time frame (Figure 5A and B). Taken together, these results demonstrate that regulation of AR expression levels cannot account for the rapid inhibitory effect of AMPK activation on AR activity that we observe.

An important step in the activation of AR is its translocation to the nucleus upon ligand binding [65]. We tested whether AMPK activity affected the localisation of the AR using nuclear-cytoplasmic fractionation of cellular lysates (Figure 5C and D). As expected, we observed increased nuclear localisation of the AR upon androgen treatment, a process which was reversed by bicalutamide. Treatment with AICAR or metformin reduced nuclear levels of AR. This suggests that AMPK might exert its inhibitory effect on AR activity by reducing its nuclear localisation following ligand binding, rather than by down-regulating its expression.

## DISCUSSION

While a large body of evidence confirms that metabolic alterations play an important role in prostate cancer, the role of AMPK in this context has remained controversial. A number of signalling pathways through which AMPK could both promote and suppress prostate cancer development and progression have been described. Using an unbiased approach, we have now identified the AR, the major driver of prostate cancer, as a downstream mediator of AMPK signalling in prostate cancer cells, adding an important new dimension to the role of AMPK in this cancer type.

To our knowledge, only one previous study has investigated the transcriptional impact of AMPK signalling in a prostate cancer context [27]. In that case, a stably transfected cell line expressing dominant-negative AMPK was used, and AMPK inhibition was found to result in differential expression of several tumour-relevant genes. Our study now extends these findings by identifying a number of additional putative AMPK targets, and by demonstrating that genes repressed by AMPK activation are overexpressed in prostate cancer specimens. Our data thus provides a resource for the field that could be used as a basis for future studies on the role of AMPK in prostate cancer.

Our data also adds further weight to a number of studies that have demonstrated off-target effects of AMPK activators, particularly metformin. While it has previously been proposed that anti-tumour effects of metformin may at least partially be mediated through AMPK-independent pathways [66, 67], our finding that approximately 90% of metformin-regulated genes are not differentially expressed after AICAR treatment demonstrates that caution must be exercised in assigning any observed effects of metformin to activation of AMPK without further validation. We believe the observed differences in dynamics of gene expression changes as well as in the extent of off-target effects between the two drugs may be due to their different mechanisms of action in activating AMPK. While AICAR is converted to AICAR 5'-Monophosphate (ZMP) within the cell, which activates AMPK by acting as an AMP mimetic [68], metformin inhibits complex I of the electron transport chain, prompting a decrease in cellular ATP/AMP ratio which then results in activation of AMPK [69]. The recent development of novel, potentially more specific activators of AMPK [70] will likely be an important step towards a better characterisation of AMPK function.

Nevertheless, we believe that our strategy of investigating gene expression changes that are shared between AICAR and metformin, two structurally unrelated AMPK activators, has yielded relevant insights into AMPK function. We have shown that biological processes enriched among our AMPK target gene signature are representative of the described function of AMPK, that our AMPK target gene signature resembles PI3K/Akt/mTor inhibition, and that predicted transcription factors shared between AICAR and metformin include known AMPK targets. All of this evidence suggests that our data is indeed representative of AMPK function.

**Figure 6:**
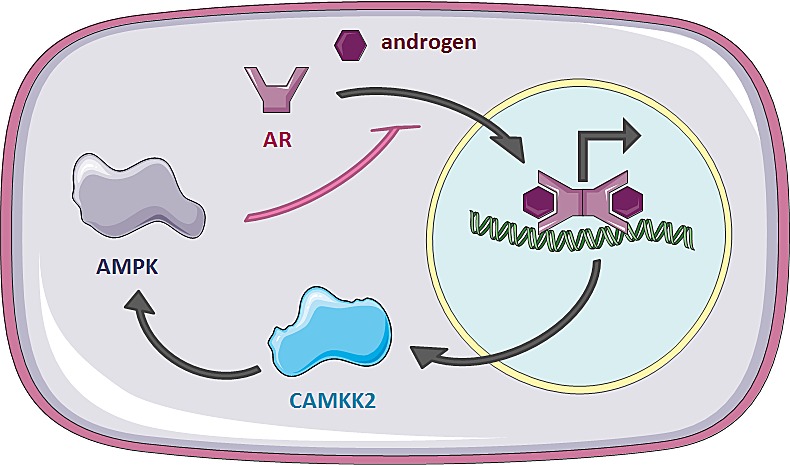
A negative feedback loop between AR and AMPK in prostate cancer cells AR translocates to the nucleus upon ligand binding to activate transcription of its target genes, including *CAMKK2*. CAMKK2 phosphorylates AMPK, resulting in increased AMPK activity following androgen stimulation. Activated AMPK, in turn, reduces nuclear localisation of the AR, thereby attenuating its activity.

Using this approach we were able to identify and validate the AR as a novel downstream mediator of AMPK signalling in prostate cancer cells. The AR drives both proliferation and anabolic pathways in prostate cancer cells [11]. In contrast, upon its activation, AMPK down-regulates anabolic pathways, stimulates catabolic pathways and reduces proliferation [9]. Notably, AR has previously been shown to regulate AMPK activity by inducing the expression of *CAMKK2* [11, 20]. Therefore, our results suggest the presence of a negative feedback loop whereby the AR increases the activity of AMPK, which in turn feeds back to reduce AR activity (Figure 6). Thus, one could speculate that AMPK functions as a safety mechanism to prevent overshooting of AR activity and resulting stimulation of anabolism and proliferation under low-energy conditions.

The exact mechanism by which AMPK is able to interfere with nuclear localisation of the AR will require further investigation. Notably, the activity of other nuclear receptors, including progesterone receptor, peroxisome proliferator-activated receptors and glucocorticoid receptor, has previously been shown to be regulated by AMPK [57, 71, 72], suggesting potential mechanisms of transcriptional regulation by AMPK that need to be explored further. First, AMPK has been shown to phosphorylate the transcriptional co-activator p300 at its serine 89 residue, thereby blocking its interaction with a number of nuclear receptors. It is thus conceivable that activation of AMPK could also disrupt interaction of p300 with AR [71]. As acetylation of the AR by p300 is thought to promote its nuclear localisation [73, 74], this mechanism could explain the decreased nuclear AR levels following AMPK activation that we observe.

Second, AMPK has been shown to stimulate the activity of p38 and JNK [57, 75-77]. These two kinases have been reported to phosphorylate AR on its serine 650 residue, promoting its nuclear export [78]. Finally, investigating links between AMPK and AR based on published data using MetaCore reveals a number of additional pathways through which AMPK may affect AR activity, including p53, PARP-1 and β-catenin signalling, hinting at the potential complexity of this regulatory system (Supplementary Figure 3). Further research will be required to unravel this intricate network of signalling pathways in order to determine the exact mechanisms of action by which AMPK interferes with AR localisation.

Interestingly, it has recently been reported that metformin can inhibit membrane-initiated androgen signalling [79], and two previous publications have shown that metformin treatment reduces AR expression levels over the course of several days [61, 62]. However, both of these effects were demonstrated to be either partially or entirely independent of AMPK. Our study thus provides the first evidence that classical AR signalling is regulated by AMPK, and suggests a mechanism involving decreased nuclear localisation of the AR. Notably, this acute inhibitory effect on AR occurs within several hours of treatment of cells with AICAR or metformin, lending further support to the notion that it represents a direct consequence of AMPK activation in the cell, rather than more indirect effects observed after long-term treatment. Considering our finding that the vast majority of the widespread metformin-induced transcriptional changes in prostate cancer cells cannot be replicated by AICAR treatment and are thus possibly independent of AMPK, it is not surprising that metformin would also affect the AR via pathways that do not involve AMPK.

In conclusion, we propose that AMPK and AR regulate each other via a negative feedback loop. A full understanding of how these two major metabolic regulators influence each other should provide important insights for the design of successful therapeutic strategies targeting prostate cancer cell metabolism.

## METHODS

### Cell culture conditions and drug treatments

Cell lines were maintained in RPMI 1640 (Invitrogen, Carlsbad, CA, USA) supplemented with 10% fetal bovine serum (Hyclone / Thermo Scientific, Waltham, MA, USA) and passaged twice per week. Androgen treatments were carried out in phenol-red free RPMI 1640 (Invitrogen) supplemented with 10% charcoal/dextrane-treated FBS (Hyclone). AICAR (Tocris Bioscience, Bristol, UK) and metformin (Sigma-Aldrich, St Louis, USA) were used at final concentrations of 0.5 mM and 2 mM, respectively. R1881 and bicalutamide (Sigma-Aldrich, St Louis, USA) were used at concentrations of 1 nM and 10 μM, respectively.

### Genome-wide expression profiling

LNCaP cells grown in RPMI with 10% FBS were treated with AICAR or metformin in five independent biological replicates. RNA was extracted after 0 h, 8 h and 24 h of treatment using RNeasy Plus Mini Kit (Qiagen, Hilden, Germany). cDNA was generated, hybridised to Illumina HumanHT-12 v4 BeadChip arrays and scanned using standard Illumina protocols. Data was analysed using R (R Development Core Team, 2010) and Bioconductor [80]. Spatial artefacts were removed using BASH [81] and HULK algorithms from the beadarray package [82]. Data was log2 transformed and quantile normalised, and differentially expressed genes were identified with a global false discovery rate of 0.01. Expression data will be deposited in Gene Expression Omnibus (GEO). Significance of gene list overlaps was tested in R using the phyper function. Heatmaps were generated using a Cancer Research UK Cambridge Institute Bioinformatics Core Facility Galaxy tool. Enrichment of gene ontology terms was tested using the Molecular Signature Database v4.0 (Broad Institute, http://www.broadinstitute.org/gsea/index.jsp). To test enrichment of gene sets of interest within publicly available prostate cancer datasets, genes were ranked from most underexpressed to most overexpressed in primary cancer compared to normal/benign tissue according to t statistic, and gene set enrichment analysis was performed using the GSEA software (Broad Institute, http://www.broadinstitute.org/gsea/index.jsp). Enriched transcription factors were identified using Ingenuity Pathway Analysis (http://www.ingenuity.com/products/ipa). Due to the extremely high number of significantly differentially expressed genes after 24 h metformin treatment, only genes with a log fold change of ± 0.65 or above were used for this analysis.

### Quantitative reverse transcription PCR

RNA from cells was isolated using the RNeasy Plus Mini Kit according to the manufacturer's instructions. cDNA was synthesized using the High Capacity cDNA Reverse Transcription Kit (Applied Biosystems, Foster City, CA, USA) following the manufacturer's instructions. qRT-PCR reactions were performed on an ABI PRISM 7900 HT Sequence Detection System. Relative gene expression was calculated according to the ΔΔCt method; *ACTB* and *SDH* were used as housekeeping genes. Details of primers used are given in Supplementary Table 2.

### Luciferase reporter assay

Cells were seeded in 48-well plates and transfected with luciferase reporter constructs MMTV-*Luc* and pRL-TK one day after seeding using Lipofectamine 2000 (Invitrogen) according to the manufacturer's instructions. To evaluate the effects of pharmacological AMPK activators, the medium was replaced with phenol-red free RPMI supplemented with charcoal-stripped serum, R1881 or vehicle and AMPK activators 6 h post-transfection. After a further 24 h, dual luciferase reporter assay was carried out using the Dual Luciferase Reporter Assay System (Promega, Madison, WI, USA) according to the manufacturer's instructions. To determine the effects of gene silencing, the constructs were co-transfected with siRNAs and the medium was replaced with phenol-red free RPMI supplemented with charcoal-stripped FBS 6 h after transfection. R1881 or vehicle was added 24 h post-transfection to allow knock-down of target genes before stimulation. Dual luciferase reporter assay was performed 40 h after stimulation with R1881.

### Generation of an androgen-responsive luciferase reporter cell line

The pGL4.16 vector from Promega was cut with XhoI and BglII, and a polylinker including ClaI, AsclI and NheI was inserted. The *Luc2CP* gene was retrieved by cutting the resulting vector with ClaI, XbaI and EagI HF and cloned into a pCSC-SP-PW-CMV-LacZ vector restricted with ClaI and XbaI. To add a selection marker, the PGK-Puromycin resistance cassette was obtained from a pSICOR-PGK-*Puro* plasmid by cutting it with BamHI and ScaI and ligated into the pCSC-*Luc2CP*, previously cut with BamHI and PmeI. The *CAMKK2* androgen-receptor binding sequence was cloned through PCR from genomic DNA from LNCaP cells (forward primer: ATGGCGCGCCGCTAGCGAATGCA TGCGGCAGTGTTCCAAT; reverse primer GGCCAGATCTGCTAGCTAAAGA AGGAAGGGAGGTGGCTGA) and inserted by recombination into the pCSC-*Luc2CP*-*Puro* vector cut with NheI.

For generation of lentiviral particles, HEK293TLA cells were transfected with the pCSC-*CAMKK2*-*Luc2CP*-*Puro* vector and packaging, envelope and reverse expression plasmids using the calcium phosphate method. Media was changed after 24 h and fresh media applied. After a further 24 h viral supernatant was filtered (45 μm pore size, Millipore, Billerica, MA) and mixed with fresh media. LNCaP cells were infected with lentiviral particles and expanded as a stable cell line (LNCaP-*CAMKK2*-Luc).

### Transient transfection of siRNAs

siRNAs against AMPK (AMPKα1: J-005027-06, J-005027-07; AMPKα2: J-005361-06, J-005361-07) were purchased from Dharmacon and transfected using Lipofectamine RNAiMAX (Invitrogen) according to the manufacturer's reverse transfection protocol. To achieve knock-down of both AMPKα subunits, we combined two siRNAs, one directed against each subunit. Two different combinations of siRNA were used to account for potential off-target effects. siAllStars (Qiagen) was used as a non-targeting control. siRNAs were transfected at a final concentration of 20 nM.

### Western blot analysis

Cells were lysed in ice-cold M-PER buffer (Pierce / Thermo Scientific) containing protease and phosphatase inhibitor cocktails and harvested by scraping. Lysates were cleared by centrifugation at maximum speed and 4°C for 10 min in a benchtop centrifuge. Protein content was quantified using a Direct Detect Spectrometer (Millipore). Samples for Western Blot were prepared by addition of 5x Laemmli buffer and boiled for 5 min. Equal amounts of protein were loaded onto 4-12% Criterion precast gels (Bio-Rad Laboratories, Hercules, CA, USA) and separated by SDS-PAGE. Proteins were transferred to nitrocellulose membranes using the iBlot™ Dry Blotting System (Invitrogen), and transfer was assessed by staining with Ponceau S (Sigma). Membranes were blocked in 5% milk (Marvel) or BSA (Sigma) in Tris-buffered saline with 0.1% Tween-20 and incubated with primary antibodies and HPRC-conjugated secondary antibodies (Dako). Detection was carried out using Western Lightning ECL Pro (Perkin Elmer). Films were scanned and quantification of intensity of bands was carried out using ImageJ. pACC Ser79(#3661), pAMPK Thr172 (40H9) and AMPKα (#2532) antibodies were from Cell Signalling Technology; β-actin (AC-40) antibody was from Sigma; lamin B (M-20) antibody was from Santa Cruz Biotechnology; β-tubulin (D66) antibody was from Abcam; AR (441) antibody was from Dako.

### Nuclear-cytoplasmic fractionation

To obtain nuclear and cytoplasmic fractions, cell pellets were resuspended in Buffer A (10 mM Hepes pH 7.9, 10 mM KCl, 1.5 mM MgCl_2_, 0.34 M sucrose, 10% glycerol) supplemented with 0.1% Triton X-100 and incubated on ice for 10 min. Nuclei were pelleted by centrifugation at 4°C and 1,300 G for 4 min. The supernatants were cleared by centrifugation to obtain cytoplasmic fractions. Nuclei were washed three times in Buffer A without Triton X-100, resuspended in Buffer B (3 mM EDTA, 0.2 mM EGTA) and disrupted by sonication. Nuclear fractions were cleared by centrifugation.

### Statistical analysis

Statistical analysis was carried out using GraphPad Prism 6. To test significant differences between groups, one-way or randomized blocks ANOVA was performed depending on the experimental design. If significance at the level of p=0.05 was reached, multiple comparisons of means were performed using Holm-Sidak test. Unless otherwise stated, all figures show means of biological replicates and error bars indicate SEM. Significance levels are indicated as follows: * p < 0.05; ** p < 0.01; *** p < 0.001.

## SUPPLEMENTARY MATERIAL


