# Supplementary material for: Transcriptomic analysis reveals inhibition of androgen receptor activity by AMPK in prostate cancer cells
Source: Oncotarget. 2014 May 21;5(11):3785–99. doi: 10.18632/oncotarget.1997 (PMC4116520; doi:10.18632/oncotarget.1997)
Supplement: Supplementary file 1 [file oncotarget-05-3785-s001.pdf]

Transcriptomic analysis reveals inhibition of androgen receptor activity by AMPK in prostate cancer cells

Supplementary Material

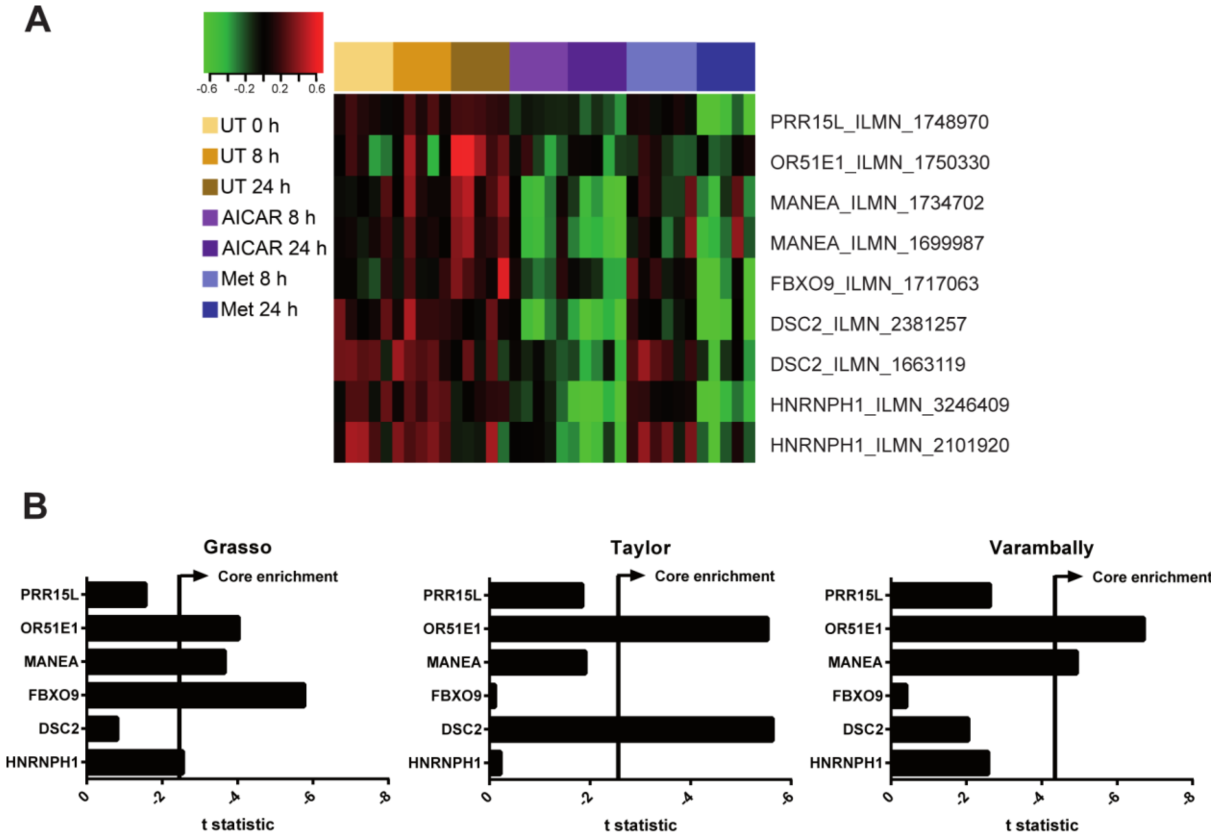

Supplementary Figure 1: Genes downregulated by AMPK are overexpressed in prostate cancer. A: Examples of six genes downregulated by AMPK activation. Heatmap shows expression of six selected genes in the expression array. **B:** AMPK repressed genes are overexpressed in prostate cancer. t statistics for the six genes in the Grasso, Taylor and Varambally datasets comparing expression in primary cancer to expression in benign/normal tissue are shown. Negative values indicate upregulation in primary cancer. Cut-offs for genes contributing to the core enrichment according to GSEA are indicated.

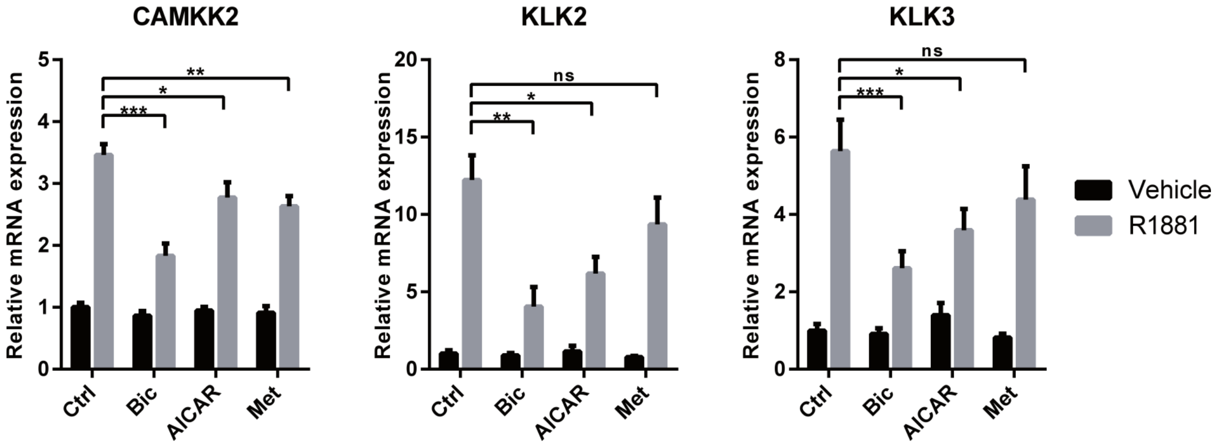

Supplementary Figure 2: Reduced R1881-induction of AR target genes in LNCaP cells following AMPK activation. To assess effects of AMPK activation on induction of endogenous AR-target genes by R1881, C4-2 cells were grown in androgen-free medium for three days and stimulated with R1881 and bicalutamide, AICAR or metformin for 12 h. mRNA expression was assessed by qRT-PCR; values are depicted relative to vehicle control. n=4 for *CAMKK2* and *KLK3*, n=3 for *KLK2*.

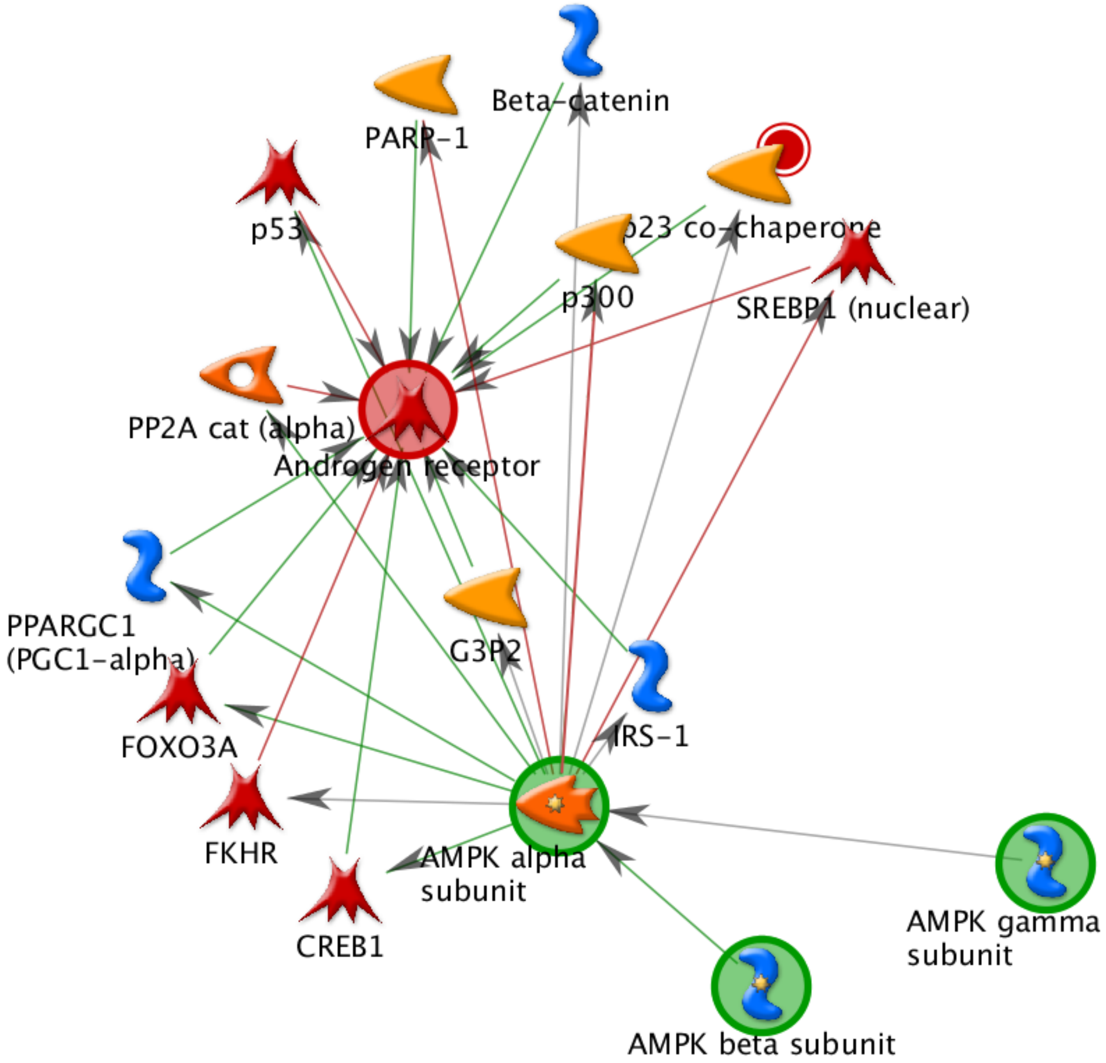

Supplementary Figure 3: A complex network of signalling pathways links AMPK to regulation of AR activity. Potential links between AMPK and AR were predicted using MetaCore. Image was generated with MetaCore, manually checked against literature and edited where required.

Supplementary Table 1: AMPK regulated genes resembles gene expression signatures associated with PI3K/Akt/mTor pathway inhibition. AMPK regulated genes were compared to gene-expression signatures associated with 1,309 bioactive small molecules using Connectivity Map 02. Table shows top 10 most significant hits.

Rank	cmap Name	p-value	Comment
**1**	5224221	< 0.00001	
**2**	thioridazine	< 0.00001	inhibits PI3K/Akt/mTOR pathway
**3**	wortmannin	< 0.00001	inhibits PI3K/Akt/mTOR pathway
**4**	LY-294002	< 0.00001	inhibits PI3K/Akt/mTOR pathway
**5**	trichostatin A	< 0.00001	
**6**	loperamide	0.00002	
**7**	thapsigargin	0.00004	
**8**	anisomycin	0.00004	
**9**	pimozide	0.00004	
**10**	perphenazine	0.00004	

Supplementary Table 2: qRT-PCR primers used in this study.

Gene	Forward Primer Sequence	Reverse Primer Sequence
**ACTB**	TTGCCGACAGGATGCAGAAGGA	AGGTGGACAGCGAGGCCAGGAT
**AR**	CTCACCAAGCTCCTGGACTC	CAGGCAGAAGACATCTGAAAG
**CAMKK2**	TGAAGACCAGGCCCGTTTCTACTT	TGGAAGGTTTGATGTCACGGTGGA
**DSC2**	GCCAAGAGAAGATGGGCTCC	TTGGGCCGTGTCAGATTGAA
**FBXO9**	GGCAGAAGCTGAGGAAGATTG	AACATCTGGAGTTGTGCCTGC
**HNRNPH1**	CAGTTCAGCGACCACGTTTG	CACCACGAATCCCTCTCCAC
**KLK2**	ATGTGTGCTAGAGCTTACTC	AAGTGGACCCCCAGAATCAC
**KLK3**	AGAAGCATTCCCAACCCTG	GTCGTGGCTGGAGTCATC
**MANEA**	CGCGGTCTTAACCTCTCCTC	TCCTTCTCCGAAACTTTGCCA
**NKX3.1**	AGAAGGCCTCCTCTTTCAGG	GCCAAGAACCTCAAGCTCAC
**OR51E1**	TTCCATACGGTTGAGCCTCT	CCAACCAGAACTGAGCCTCT
**PRKAA1**	TTGAAACCTGAAAATGTCCTGCT	GGTGAGCCACAACTTGTTCTT
**PRKAA2**	ACCAGGTGATCAGCACTCCA	TCTCTTCAACCCGTCCATGC
**PRR15L**	CTTGCTTCCCGAGTCTCACC	GGGCTTTCTGTAATCCCGAC
**SDH**	TGGGAACAAGAGGGCATCTG	CCACCACTGCATCAAATTCATG
**TMPRSS2**	CCATTTGCAGGATCCGTCTG	GGATGTGTCTTGGGGAGCAA
